# Antibiotic prescribing and non-prescribing in nursing home residents with signs and symptoms ascribed to urinary tract infection (ANNA): study protocol for a cluster randomized controlled trial

**DOI:** 10.1186/s12877-020-01662-0

**Published:** 2020-09-11

**Authors:** Jeanine J. S. Rutten, Laura W. van Buul, Martin Smalbrugge, Suzanne E. Geerlings, Debby L. Gerritsen, Stephanie Natsch, Philip D. Sloane, Ruth B. Veenhuizen, Johannes C. van der Wouden, Cees M. P. M. Hertogh

**Affiliations:** 1 Department of Medicine for Older People, Amsterdam University Medical Center, Vrije Universiteit, Van der Boechorststraat 7, 1081 BT Amsterdam, the Netherlands; 2 Department of Internal Medicine, Infectious Diseases division, Amsterdam University Medical Center, University of Amsterdam, Amsterdam, the Netherlands; 3 Department of Primary and Community care, Radboud Institute for Health Sciences, Radboudumc Alzheimer Center, Radboud University Medical Center, Nijmegen, the Netherlands; 4 Department of Pharmacy, Radboud University Medical Center, Nijmegen, the Netherlands; 5 Department of Family Medicine, School of Medicine, and the Cecil G. Sheps Center for Health Services Research, University of North Carolina, Chapel Hill, NC USA

**Keywords:** Urinary tract infection, Nursing homes, Antibiotic prescribing, Antibiotic stewardship, Decision support, Randomized controlled trial

## Abstract

**Background:**

Antibiotic overprescribing for suspected urinary tract infection (UTI) in nursing homes (NHs) is common. Typical clinical scenarios in which antibiotics are inappropriately prescribed include response to nonspecific signs and symptoms and/or a positive urine test in the absence of symptoms referable to the urinary tract. These and other scenarios for inappropriate antibiotic prescribing were addressed in a recent international Delphi study which resulted in the development of a decision tool for the empiric treatment of UTI in frail older adults. The aim of the current study is to implement this decision tool, by integrating it into the electronic health record (EHR) and providing education on its content and use, and to evaluate its effect on appropriate antibiotic prescribing. An additional aim is to evaluate the quality of the intervention and the implementation process.

**Methods:**

A cluster Randomized Controlled Trial (cRCT) is conducted in sixteen NHs and aims to include 897 residents diagnosed with suspected UTI. NHs in the intervention group use the EHR-integrated decision tool, and receive education for physicians and nursing staff; in the control group care as usual is provided. Data is collected through case report forms within the EHR at the day of diagnosis and at 3, 7, and 21 days thereafter. The primary outcome is appropriate antibiotic prescribing for suspected UTI at the day of diagnosis. Secondary outcomes include the course of symptoms, alternative diagnoses, treatment changes, complications, hospitalization, and mortality. Data on total antibiotic prescribing are additionally collected in the participating NHs 12 months before and during the study. Finally, the process evaluation combines cRCT data with questionnaires and qualitative interviews with NH professionals.

**Discussion:**

This is the first cRCT to evaluate the recently developed, international decision tool for empiric treatment of suspected UTI in NH residents. Study findings will elucidate the effect of the intervention on appropriate antibiotic prescribing for suspected UTI, and provide insight into the applicability of the decision tool in NHs in general and in specific subgroups of NH residents. With this study we aim to contribute to antibiotic stewardship efforts in long-term care.

**Trial registration:**

The ANNA study was registered at the Netherlands Trial Register on 26 February 2019, with identification number NTR NL7555.

## Background

Urinary tract infections (UTIs) are common among nursing home (NH) residents and account for the largest share of antibiotic prescribing in this setting [1]. Diagnosing UTIs is challenging in the NH population because presenting symptoms are often not typical and cognitive disabilities can impede communication of experienced complaints. This diagnostic uncertainty, in combination with other factors such as expectations of residents and their family members, drives antibiotic prescribing to be ‘better safe than sorry’ [2].

A substantial part of the antibiotics prescribed for suspected UTI in NHs, however, is considered inappropriate [3]. There are two common situations in which this is the case. The first is for nonspecific signs and symptoms (eg, mental status change or behavioral problems); these are often not attributable to UTI but to one of many other possible causes [4]. Second, antibiotics are frequently prescribed in response to a positive urine test (eg, a dipstick test) [5–8]. There is a high a priori chance of positive test results due to the up to 50% prevalence of asymptomatic bacteriuria in the NH setting, a condition for which antibiotics are not indicated [9–12]. Urine testing is therefore considered only useful to rule out UTI when the results are negative, but otherwise should not influence treatment decisions [4].

Inappropriate antibiotic use is an important problem. For the individual, inappropriate antibiotic use is associated with an increased risk of unnecessary exposure to side-effects and drug interactions. In addition, inappropriate antibiotic use may be accompanied by under-treatment of other conditions, if no further assessment is done to look for alternative causes that may underlie the signs and symptoms ascribed to UTI. On the societal level, inappropriate antibiotic use plays an important role in the development of antibiotic resistance [1].

In order to improve appropriateness of antibiotic prescribing in NHs, a decision tool to support medical decision making in frail older adults with a possible UTI was recently developed in an international Delphi study [4]. Here we describe the protocol of a cluster Randomized Controlled Trial (cRCT) in which this decision tool is implemented in NHs by incorporating it into the electronic health record (EHR), and by educating professionals on its content. The first objective of the study is to evaluate whether this results in more appropriate antibiotic prescribing for NH residents with suspected UTI. In addition, we evaluate the quality of the intervention and the implementation process.

## Methods

The Antibiotic prescribing and Non-prescribing in Nursing home residents with signs and symptoms Ascribed to urinary tract infection (ANNA) study aims to evaluate, in a cRCT, the effect of an EHR-integrated decision tool in combination with education, on the appropriateness of antibiotic prescribing for NH residents with suspected UTI. In addition, it aims to evaluate the quality of the intervention and the implementation process in a process evaluation.

### Cluster randomized controlled trial

Table 1 provides a schematic overview of resident enrollment, intervention, and data collection (ie, SPIRIT schedule).

**Table 1:** SPIRIT schedule of resident enrollment, intervention, and data collection

	Allocation	Enrolment	Data collection				Close-out
TIMEPOINT	Before study onset	T0 (index)	T0 (index)	T1 (day 3)	T2 (day 7)	T3 (day 21)	After completion data-collection
**ENROLMENT**							
***Eligibility screen***		X					
***Informed consent***	X						
***Allocation***	X						
**INTERVENTIONS**							
***UTI treatment decision tool in EHR***		X	X				
***Education for physicians & nursing staff***	X						
**ASSESSMENTS**							
***Patient characteristics***			X				
***Primary outcome*** *(appropriate antibiotic prescribing)*			X				
***Secondary outcomes*** *(course of symptoms, alternative diagnoses, changes in treatment policy, hospitalization, morality)*			X	X	X	X	
***Total antibiotic prescribing***							X

*Abbreviations: UTI* Urinary tract infection*, EHR* Electronic health record

#### Study design and setting

A cRCT is conducted with NHs as the unit of randomization. Although our sample size calculation (see below) required the inclusion of fifteen NHs in the study, a total of sixteen NHs across the Netherlands participate in the study. Data collection has started in March 2019 and lasts approximately 1 year.

Residents of Dutch NHs typically reside on somatic wards (ie, for residents with mainly physical conditions); psychogeriatric wards (ie, for residents with cognitive disabilities such as dementia); or geriatric rehabilitation wards. Due to high turn-over rates, geriatric rehabilitation wards are excluded from the study. Unique to Dutch NHs is that medical care is mainly provided by specialized ‘elderly care physicians’. Elderly care physicians are employed by, and have their main site of practice in the NH, although they increasingly also provide care for frail older persons living at home [13]. Other providers of medical care, similarly employed by the NH, may include physicians with other specializations (eg, geriatrics, family medicine), junior doctors, elderly care physicians in training, and nurse practitioners. Where the word ‘physician’ is used in this paper, we refer to all these medical care providers.

#### Informed consent procedure

Before study onset, all residents (or their representatives, in case of legal incapacity) of participating NH wards received written information about the study and an informed consent form. During the study, all newly admitted residents similarly receive this information and informed consent form. Consent is asked for participation in the study in case a resident develops a suspected UTI during the study period and is eligible for inclusion (ie, pre-emptive consent). Consent is also asked for using their data in ancillary studies. In addition to the written information, NH physicians provide residents with verbal information on the study during regular contact moments.

#### In- and exclusion criteria

NH residents are included in the study if they are diagnosed with suspected UTI and provided informed consent. Excluded are residents who are already taking antibiotics or have taken antibiotics in the previous 7 days, and residents who do not wish to be treated with antibiotics in case of a UTI.

#### Sample size

We consider an increase of at least 20% appropriate antibiotic prescribing for suspected UTI to be clinically relevant (ie, based on previous research from our group, from 66% in the control group to 86% in the intervention group) [14]. To detect this difference with 80% power and a significance level of 5%, 72 cases of antibiotic prescribing for UTI would be required in each group [15]. Based on previous study data [14], we expect that antibiotics will be prescribed in 91% of cases of suspected UTI in the control group, and that our intervention has the potential to reduce this to 62% of suspected UTI in the intervention group. Consequently, 79 cases of suspected UTI are required in the control group to include 72 antibiotic prescriptions, and up to 116 in the intervention group.

Dutch NHs possess approximately 150 beds, per location, on average. Dutch surveillance studies report an incidence rate of 87 UTIs per 150 beds per year [16]. Based on prior, comparable research [14], we estimate that 70% of the residents (or their representatives in case of legal incapacity) provides informed consent to participate in the study, which converts to 61 recruited residents per 150 beds per year for the present study.

Corrected for clustering within NHs, we need (79 × 4.6=) 363 cases in the control group and (116 × 4.6=) 534 in the intervention group (design effect: 1 + ((cluster size − 1) x intraclass correlation coefficient) = 1 + ((61–1) × 0.06) = 4.6). The estimate of the intraclass correlation coefficient is based on Campbell et al and prior study data [14, 17].

To include 363 cases over a period of 12 months, (363/61) 6 NHs are required in the control group. To include 534 cases over a period of 12 months, (534/61) 9 NHs are required in the intervention group.

**Fig. 1:**
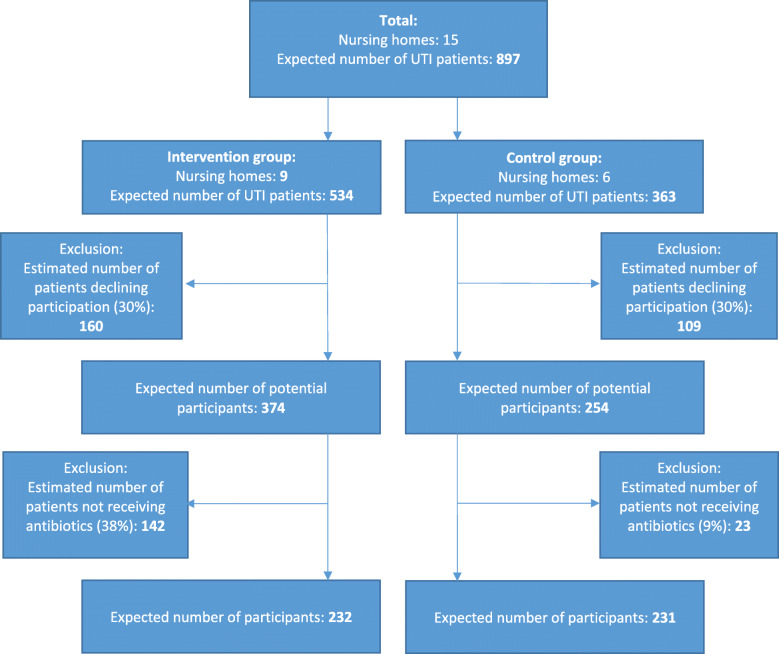
Flow diagram with the intended number of participants

Figure 1 provides a flow diagram with the intended number of participants.

#### Intervention

##### Decision tool

When a physician reports pre-structured clinical information in the EHR of residents with a suspected UTI and who provided informed consent, the decision tool automatically generates a treatment advice. The physician is free to deviate from the treatment advice and remains responsible for the treatment decision. All physicians receive a pocket card of the decision tool for situations without access to the EHR (and therefore to the decision tool).

##### Physician education

Physician education consists of: 1) a one-hour interactive presentation about the content of the decision tool, provided by the research team, and 2) a role play to learn how to deal with pressure to prescribe antibiotics from residents, their family or nursing staff, and on how to train nursing staff on the content of the decision tool.

##### Nursing staff education

The complete nursing staff is offered a 6-min video about dealing with suspected UTI in NH residents (based on a training module developed by prof. Sloane and his research group, University of North Carolina at Chapel Hill). In this video, particular attention is paid to standardized assessment of residents with suspected UTI and to other common causes of non-specific signs and symptoms. A part of the nursing staff (ie, at least one nurse per ten residents) additionally completes a 20-min e-learning to become ‘experts’ with sufficient knowledge for education of other nursing staff. In the e-learning, the video topics are discussed in more detail. Furthermore, attention is paid to how to deal with pressure from residents or their family asking for urine analysis or an antibiotic prescription. After finishing the e-learning, ‘experts’ receive a pocket card with a summary of the e-learning content. Finally, information material on actively monitoring residents who are not prescribed antibiotics, is provided to nursing staff for distribution to residents and/or their family members.

#### Control group

In the control group, care as usual is provided without any restrictions.

#### Randomization and blinding

A block randomization procedure was performed by an independent individual using randomization software. Since physicians in the intervention group use the decision tool, and the research team provides education to physicians and nursing staff, blinding of NH professionals and the research team was not feasible.

#### Data collection

##### Research application

Both in the intervention and control group, data is collected through a research application integrated in the EHR. The application starts automatically when a physician enters the diagnosis suspected UTI in the EHR of a resident that provided informed consent. This reduces chances of missing potential inclusions. To foster the collection of correct and complete data, the research application is designed in such a way that only realistic values can be entered and that case report forms (CRFs) can only be submitted if all questions regarding symptomatology and treatment have been answered.

##### Collected data

The period of data collection for each participant is 3 weeks from the day of diagnosis. The first CRF that pops up comprises in- and exclusion criteria for participation in the study. If a resident is eligible to participate, a new CRF (T0) appears that includes resident characteristics (ie, sex and age), co-morbidities (eg, dementia, urinary tract abnormalities), and index consultation data such as signs and symptoms (specific, nonspecific and systemic), additional diagnostics (eg, urine analysis) and treatment (eg, whether antibiotics were started or not). The application automatically generates follow-up CRFs to be completed on day 3 (T1), 7 (T2) and 21 (T3). In addition to EHR-generated data, anonymous data on total antibiotic prescribing in the NH is collected in the 12 months before and during the study.

##### Outcomes

The primary study outcome is appropriate antibiotic prescribing for suspected UTI at index consultation (yes/no), ie, prescribing in compliance with the treatment advice generated by the decision tool. Secondary study outcomes include the course of symptoms after the index consultation (ie, recovery, unchanged or deterioration), alternative diagnoses after assessment, changes in treatment policy (eg, start/stop antibiotics, adjustments in dose, type or duration) and motivation for these changes, complications, hospitalization, mortality and total antibiotic prescribing on the NH level.

##### Analysis

The primary analysis assesses the difference between the intervention and the control group in appropriate antibiotic prescribing for suspected UTI, treated with antibiotics, at index consultation. A three level logistic regression model is used to account for variation at the NH/physician/resident level. lf there is no indication of random effects at the physician level, the three level logistic multilevel regression model is reduced to a two level model. Multilevel regression modeling is similarly used to compare secondary study outcomes between the two groups (linear or logistic, as appropriate). A second-order penalized quasi-likelihood estimation procedure is applied, using MLwiN software. Data on total antibiotic prescribing in the 12 months prior to the start of the study, and during the study period is explored descriptively in order to describe the potential impact of adjusted antibiotic prescribing for UTI on total antibiotic prescribing within intervention NHs, compared to control NHs.

### Process evaluation

The process evaluation includes investigation of the quality of the intervention (decision tool, physician education and nursing staff education) and the implementation process, including barriers and facilitators that are involved. To this end, quantitative (cRCT data, questionnaires for NH professionals) and qualitative (semi-structured interviews with NH professionals) approaches are combined. Among professionals participating in the control group, we use similar resources to evaluate potential influences of participation in the current study or in other initiatives addressing appropriate prescribing, on antibiotic prescribing behavior.

Quantitative cRCT data is collected on several indicators for intervention quality, such as compliance with the treatment advice generated by the decision tool, and on motivations for deviation from the treatment advice. Questionnaires and semi-structured interviews are used to collect additional data on intervention quality, data on the implementation process, and data on barriers and facilitators for the implementation and use of the intervention. Regarding the latter, special attention is paid to the applicability of the decision tool in residents with limited abilities for self-report of symptoms, such as residents with dementia and residents with communicative disabilities (eg, stroke patients with aphasia). Questionnaires are distributed to all nursing staff and physicians of the participating locations. Interviews are held with a varied selection of these professionals (i.e. from very actively to poorly involved professionals). For the interviews we use a topic list based on the consolidated Framework for Implementation Research (cFIR) of Damschroder [18].

Univariate and multivariate analyses are performed to study associations between (non)adherence to the treatment advice and relevant resident characteristics (eg, dementia, incontinence, aphasia). ln addition, we investigate whether the level of implementation of the intervention is associated with adherence to the treatment advice. Finally, we describe facilitators and barriers that may explain differences in the use and implementation of the intervention. These facilitators and barriers are extracted from descriptive questionnaire analysis and from deductive thematic analysis of interviews.

## Discussion

This protocol paper described the design of a cRCT to evaluate whether an EHR-integrated decision tool in combination with education results in an increase in appropriate antibiotic prescribing for NH residents with suspected UTI. The decision tool under investigation was recently developed in an international Delphi procedure [4]. To the best of our knowledge, this is the first RCT to evaluate its use, and in addition, the first to evaluate the implementation of an electronic decision support for UTI in this setting. The studied intervention has the potential to improve diagnostic evaluation and treatment for suspected UTI in NH residents, and so contribute to the increasing antibiotic stewardship efforts in this setting.

### Reflection on study design

#### Informed consent procedure

The pre-emptive informed consent procedure applied in our study implies that all residents of participating NH wards are asked for informed consent, whereas only those who develop a suspected UTI during the study period become eligible for participation. This method allows physicians to use the decision tool and to start collecting data immediately after they consider a UTI. This would not be possible if physicians would have to ask for consent at the time of diagnosis, as legislation requires allowing time to reflect before deciding on study participation. The disadvantage of this pre-emptive informed consent procedure is that it is very time-consuming and puts a burden on residents, and/or their representatives, that will never become eligible to participate in our study.

#### Inclusion criteria

We aim to include participants that represent the general NH population and therefore apply broad inclusion criteria. Whereas people with dementia are often excluded from research [19], in our study it is especially important to include this group. Previous research showed that inappropriate antibiotic treatment for UTI is especially common in NH residents with dementia, as a consequence of communication issues and a high prevalence of nonspecific symptoms such as mental status change [3]. The vulnerability of this patient population emphasizes the importance of considering other causes for nonspecific signs and symptoms, and of introducing appropriate management. An important element of the process evaluation of the current study is to assess applicability of the decision tool in residents with dementia. If this would turn out to be limited, the results may provide clues for optimizing the decision tool for this specific population.

#### Intervention

In line with reports on computerized decision support systems for antibiotic prescribing in other health care settings, we believe that our intervention can be powerful in promoting appropriate antibiotic prescribing in the NH setting [20, 21]. The benefit of automatically generated treatment advice may be further reinforced by the fact that all physicians receive training on the content and background of the decision tool. Another strength of our intervention is its multidisciplinary character. The intervention is not solely oriented towards physicians, but also towards nursing staff. Nursing staff plays an important role in diagnosing UTI, as they observe and communicate signs and symptoms and may perform urine tests. In addition, they play an important role in communicating treatment decisions to residents and/or their family members. We therefore believe that involving nursing staff is a prerequisite for implementing changes regarding UTI diagnosis and management.

A potential pitfall is that access to a device with the EHR is needed at the time of treatment decision making in order to use the electronic decision support. If this is not the case, physicians may make treatment decisions before they received treatment advice. We anticipate this problem by providing pocket cards displaying the decision tool for these situations.

#### Data collection

Both in the intervention and the control group, physicians structurally report signs and symptoms of suspected UTI in electronic CRFs, for the study purposes. This may be considered an intervention in itself, as it provides insight into one’s treatment decision-making process, and additionally may promote behavior change due to the awareness of ‘being observed’ (ie, the Hawthorne effect) [22]. This would be in line with our previous study that showed a positive effect of data collection activities on the appropriateness of antibiotic prescribing decisions, which faded out over time [14]. In the current study we address this possible effect in the process evaluation (ie, the influence of study data registration is considered in interviews with physicians from both the intervention and control group) and by evaluating prescribing data (ie, a possible Hawthorne effect may be reflected by a change in antibiotic prescribing after study onset in both study groups, as compared with prescribing data prior to study onset).

### Reflection on study context

#### Revised guideline on UTI

We originally intended to evaluate the decision tool before its content would become usual care. However, during the final preparations of our study, the decision tool was incorporated in the revised guideline on UTI of the Dutch Association of Elderly Care Physicians (Verenso). From that moment on, the decision tool has been accessible for all physicians, including the physicians in our control group. Nonetheless, it is widely known that implementation of guidelines takes a substantial amount of time. In addition, earlier studies showed that 30 to 40% of the patients are not treated according to the current scientific evidence [23]. As the implementation of the decision tool represents a true paradigm shift (ie, the construct ‘UTI’ has been altered, which requires a different approach to nonspecific signs and symptoms and asks for changing habits with regard to urine testing) [4, 11], we anticipate that the implementation of this particular UTI guideline is even more challenging. The intervention that we evaluate in the current study may provide the support that is needed to provoke the much needed change in diagnosis and management of UTI.

#### External antibiotic stewardship efforts

Over the past few years there has been increased scientific and political attention for antibiotic stewardship in the long-term care setting in the Netherlands [24]. This has triggered a wide range of activities by several parties, focused on improving infection prevention and appropriate antibiotic use in NHs. The above mentioned UTI guideline revision is one of these activities. In the process evaluation, we establish if and to what extent the NHs in our study have participated in these activities. This is highly important for the interpretation of the current study results, as some activities might influence our primary study outcome: appropriate antibiotic prescribing for suspected UTI.

#### Decision tool not validated

Although the decision tool is incorporated into the aforementioned revised UTI guideline, it has not been validated to date. This implies that potential consequences of using the decision tool, in terms of disease course and resident safety, have not yet been investigated. Furthermore, after publication of the revised guideline, professionals raised several questions about the decision tool, in particular about its applicability to residents with dementia. Our study is of great importance to provide clarity in these issues.

This is the first cRCT to evaluate the recently developed, international decision tool for empiric treatment of suspected UTI in NH residents. Implementation of the decision tool includes integration into the EHR combined with multidisciplinary-oriented education on its content and use. Study findings will elucidate the effect of the intervention on appropriate antibiotic prescribing for suspected UTI, and provide insight into the applicability of the decision tool in NHs in general and in specific subgroups of NH residents, such as residents with dementia. With this study we aim to contribute to antibiotic stewardship efforts in the long-term care setting.

## Data Availability

The datasets generated and/or analyzed during the current study will be deposited in the repository PURE within a maximum of 3 months after publication of study results. The dataset(s) involved will be anonymised/pseudonymised and can be accessed under restrictions.

## Abbreviations

EHR: Electronic Health Record
NH: Nursing Home
cRCT: Cluster Randomized Controlled Trial
UTI: Urinary Tract Infection
